# Habit

**Published:** 1861-10

**Authors:** 


### HABIT.

Burke relates that, for a long time, he had been under the necessity of frequenting a certain place every day, and that, so far from finding a pleasure in it, he was affected with a sort of uneasiness and disgust; and yet, if by any means he passed by the usual time of going thither, he felt remarkably uneasy, and was not quieted until he was in his usual track.

Persons who use snuff soon deaden the sensibility of smell, so that a pinch is taken unconsciously, and without any sensation being exerted thereby, sharp though the stimulus may be.

After a series of years' winding up a watch at a certain hour, it becomes so much a routine as to be done in utter unconsciousness; meanwhile, the mind and body, also, are engaged in something wholly different.

An old man is reported to have scolded his maid-servant very severely, for not having placed his glass in the proper position for shaving. "Why sir," replied the girl, "I have not done so for months, and I thought you could shave just as well without it."

We all are creatures of habit; and the doing of disagreeable things may become more pleasant than omissions: showing to the young the importance of forming correct habits in early life, to the end that they may be carried out without an effort, even although, at first, it may have required some self-denial, some considerable resolution, to have fallen into them.

But, if doing disagreeable things does, by custom, become more pleasurable than their omission, then the doing right, because we love to do what is right, becomes a double pleasure to the performer, in the consciousness that, while he is yielding allegiance to his Maker, he benefits his fellow man, and cannot get out of the habit of well-doing without an effort and a pang. Thus are the truly good hedged round about, and are more confirmed in their good doing, and its practice becomes easier and more delightful, the longer they live, helping them to go down to the grave "like as a shock of corn cometh in his season."

But if there is something in the fixedness of good habits which binds us to them, there is the same thing as to the evil. Thus it is, that, when a man has arrived at the age of forty-five years, he seldom changes his opinions or his practices, which, if they are evil, become more and more fixed. Thus, what a man believes and practices at forty-five, he is likely to believe and practice till he dies, and there is small hope of his conversion to different views or different deeds, and the Ethiop's skin or the leopard's spots are his forever. The man, therefore, who is not a Christian by principle and profession and practice, at that age, should regard his condition " with fear and trembling," for it is most likely that he never will be one.

---

*Springfield Republican* has become famous of late, by the many extracts of a wholesome practical nature taken from its editorial columns, the writer being an educated physician; among others is an item on The Trials of Married Life:

" Married life has its trials and its sorrows. Tempers may prove incompatible, and call for forbearance. Fortune may be chary of its favors, and enforce self-denial. Children may be ungrateful, and sting the poor heart that has pillowed them. Sickness may come, and haunt a household for years. But ask the poor man, struggling along with his debts, and the weary woman, toiling early and late, accomplishing the ruin of her beauty and her buoyancy, if they would be placed apart, could competence be given them, and all their trials be brought to an end. The answer would be: "There is something sweeter in this companionship of suffering, than anything the world can offer from its storehouse of joys outside of it, and something which would make even severer trials than ours only iron bands to draw us more firmly together."

